# Advanced glycation end products promote ChREBP expression and cell proliferation in liver cancer cells by increasing reactive oxygen species

**DOI:** 10.1097/MD.0000000000007456

**Published:** 2017-08-18

**Authors:** Hanbei Chen, Yakui Li, Yemin Zhu, Lifang Wu, Jian Meng, Ning Lin, Dianqiang Yang, Minle Li, WenJin Ding, Xuemei Tong, Qing Su

**Affiliations:** a Department of Endocrinology, Xinhua Hospital; b Department of Biochemistry and Molecular Cell Biology, Shanghai Key Laboratory for Tumor Microenvironment and Inflammation, Key Laboratory of Cell Differentiation and Apoptosis of Chinese Ministry of Education, Shanghai Jiao Tong University School of Medicine, Shanghai; c State Key Laboratory of Cellular Stress Biology, Innovation Center for Cell Signaling Network, School of Life Sciences, Xiamen University, Xiamen, Fujian; d Department of Gastroenterology, Xinhua Hospital, Shanghai Jiao Tong University School of Medicine, Shanghai, China.

**Keywords:** advanced glycation end products, carbohydrate responsive element-binding protein, reactive oxygen species

## Abstract

The aim of the study was to elucidate the mechanism by which advanced glycation end products (AGEs) promote cell proliferation in liver cancer cells.

We treated liver cancer HepG2 cells with 200 mg/L AGEs or bovine serum albumin (BSA) and assayed for cell viability, cell cycle, and apoptosis. We performed real-time PCR and Western blot analysis for RNA and protein levels of carbohydrate responsive element-binding protein (ChREBP) in AGEs- or BSA-treated HepG2 cells. We analyzed the level of reactive oxygen species (ROS) in HepG2 cells treated with AGEs or BSA.

We found that increased S-phase cell percentage and decreased apoptosis contributed to AGEs-induced liver cancer cell proliferation. Real-time PCR and Western blot analysis showed that AGEs stimulated RNA and protein levels of ChREBP, a transcription factor promoting glycolysis and maintaining cell proliferation in liver cancer cells. Intriguingly, the level of ROS was higher in AGEs-treated liver cancer cells. Treating liver cancer cells with antioxidant *N*-acetyl cystein (NAC) partly blocked AGEs-induced ChREBP expression and cell proliferation.

Our results suggest that the AGEs-ROS-ChREBP pathway plays a critical role in promoting ChREBP expression and liver cancer cell proliferation.

## 1 Introduction

Advanced glycation end products (AGEs), products of the nonenzymatic binding of free reducing sugars and reactive carbonyls to proteins, are formed within the body during homeostasis.^[1,2]^ Rates of AGEs formation have been linked to redox balances.^[3,4]^ Excessive accumulation of AGEs occurs in pathological conditions such as hyperglycemia.^[3,4]^ Cell surface receptors for AGEs include the receptor for advanced glycation end products (RAGE), oligosaccharyl transferase-48 (OST-48), scavenger receptors types I and II, and galectin-3 and 80K-H phosphoprotein.^[3,4]^ Interactions between AGEs and their receptors evoke oxidative stress and inflammatory reactions in a variety of cells, thereby being involved in different aging- or diabetes-associated disorders, including cardiovascular disease, chronic kidney disease, osteoporosis, Alzheimer's disease, and cancer.^[5–12]^

The role of AGEs in the initiation and progression of cancer is drawing more and more attention. AGE treatment of different cancer cell lines promotes cell growth, migration, and invasion.^[13–17]^ Given the increased cancer-related mortality in patients with diabetes mellitus, these studies indicate that AGEs may represent a potential mechanistic link between hyperglycemia and cancer. Our group has previously reported that AGEs increase colorectal and liver cancer cell proliferation.^[15]^ In colorectal cancer cells, the AGEs-RAGE-ChREBP signaling plays an important role in enhancing cell proliferation.^[15]^ However, the mechanism by which AGEs promote cell proliferation in liver cancer cells remains to be elucidated.

Oxidative stress is an imbalance between reactive oxygen species (ROS) and antioxidant defense system.^[18]^ ROS at certain levels act as signal molecules to stimulate cell proliferation, apoptosis, and gene expression.^[18]^ Excessive ROS produce oxidative stress that plays an important role in the development of diabetes and cancer.^[19,20]^ High AGEs levels contribute to the increased generation of ROS in diabetic patients.^[19,20]^ The binding of AGEs to RAGE induces ROS production, which in turn modulates activation of protein kinase C, mitogen-activated protein kinases, and various cytokines and transcription factors such as FOXO, Nrf2, AP-1, and NF-κB.^[3,19]^ Moreover, persistent oxidative stress can lead to uncontrollable cell proliferation and neoplasm formation.^[20]^ ROS are highly reactive and can directly damage cellular constituents such as DNA, lipids, and proteins.^[18,20]^ ROS can also regulate expression of many genes which encode proteins involved in cell-cycle regulation, cell proliferation, and apoptosis.^[18,20]^ ROS have been known to play important roles in hepatocarcinogenesis. Different inducers for liver cancer including hepatitis viral infections, carcinogens, toxins, and steroid hormones have a common denominator ROS.^[21]^

ChREBP is an important glucose-responsive transcription factor for genes encoding key enzymes in glycolysis and lipogenesis such as L-type pyruvate kinase (L-PK), acetyl-CoA carboxylase (ACC), fatty acid synthase (FAS), and stearoyl-CoA desaturase-1 (SCD1) in metabolic tissues and cancer cells.^[22–24]^ In addition to the transcriptional activator role, ChREBP also functions as a transcriptional repressor which decreases the expression of many genes including phosphoenolpyruvate carboxykinase (PEPCK), glucose-6-phosphatase (G6Pase), solute carrier family 6 member 9 (SLC6A9), and tribbles homolog 3 (TRIB3).^[25]^ Glucose not only enhances ChREBP mRNA and protein expression, but also increases the activity of ChREBP by promoting its nuclear translocation and DNA-binding activity.^[22,23,26]^ In addition to the canonical ChREBP isoform (ChREBP-α), the newly identified isoform ChREBP-β transcribed from a different promoter is also regulated by glucose.^[27]^ Interestingly, mRNA levels of ChREBP are altered in obese adolescents with prediabetes or early type 2 diabetes.^[28]^ Besides its function in regulating metabolism in the diabetic condition, ChREBP promotes aerobic glycolysis, anabolism, and proliferation in colorectal and liver cancer cells.^[24]^ We have recently reported that AGEs-induced ChREBP upregulation and activation contributes to increased cell proliferation in colorectal cancer cells in response to AGEs.^[15]^

In the present study, we aimed to find out the underlying mechanism of AGEs in regulating liver cancer cell proliferation. We treated liver cancer cells with AGEs under different glucose conditions. 0 mM, 5.6 mM, and 25 mM glucose represented no glucose, physiological concentration of glucose and high glucose conditions, respectively. We found that AGEs induced liver cancer cell proliferation by increasing S-phase population and inhibiting apoptosis. AGEs treatment increased ROS production in liver cancer cells. The antioxidant NAC partly blocked AGEs-induced ChREBP expression and cell proliferation, suggesting that the AGEs-ROS-ChREBP pathway played a critical role in promoting liver cancer cell proliferation. Our findings suggest that the AGEs-ROS-ChREBP pathway might be new targets for treating liver cancer in diabetic patients.

## 2 Materials and methods

Ethical approval was not necessary because no patients’ information was collected according to Ethics Committee of Xin Hua Hospital Affiliated to Shanghai Jiao Tong University School of Medicine.

### 2.1 Cell culture

Human hepatocellular carcinoma HepG2 cells were cultured at 37°C under humidified 5% CO_2_ atmosphere in Dulbecco's Modified Eagle's Medium (DMEM) supplemented with 0 mM, 5.6 mM, or 25 mM glucose, 10% fetal bovine serum (FBS), 100-unit penicillin/mL, 100 μg streptomycin/mL, 1 mM sodium pyruvate, 50 μmol/L β-mercaptoethanol, and 2 mM L-glutamine.

### 2.2 Preparation of AGEs

AGEs were prepared as described previously.^[15]^ All incubations were performed under sterile conditions. AGEs were prepared by incubating BSA (50 mg/mL, Sigma) with D-glucose (0.5 M, Sigma) in phosphate buffer (0.2 M, pH 7.4) for 8 weeks. After incubation, unbound sugar was removed by extensive dialysis against PBS (0.2 M, pH 7.4). As a negative control, BSA (50 mg/mL) was incubated without D-glucose under the same conditions for 8 weeks. AGEs were tested for endotoxin using limulus amebocyte lysate (LAL) reagent (Associates of Cape Cod). If the endotoxin was less than 15 EU/L, the preparations were considered to be successful.

### 2.3 Cell viability, cell cycle, and apoptosis analysis

For cell viability analysis, HepG2 cells cultured in different glucose conditions were treated with AGEs or BSA for 72 hours. Cell viability was performed by the MTS assay (Promega).

Cell cycle was estimated using the BrdU APC Flow Kit (BD Pharmingen) as described previously.^[15]^ HepG2 cells cultured in glucose-free DMEM were treated with AGEs or BSA for 24 hours.

For cell apoptosis analysis, HepG2 cells cultured in glucose-free DMEM were treated with AGEs or BSA for 24 hours. Cell apoptosis was performed by using FITC Annexin V Apopotosis Detection Kit (BD Pharmingen) as described previously.^[15]^

### 2.4 Nuclear and cytosolic fractionation and western blot analysis

AGEs were added to HepG2 cells cultured in DMEM supplemented with 0 mM or 25 mM glucose and cells were cultured for another 24 hours. Nuclear and cytosolic fractionation and western blot analysis was performed as described previously.^[15]^ The following primary and secondary antibodies were used: anti-ChREBP (Novus), and anti-Tubulin (Sigma) antibodies, and secondary peroxidase labeled anti-IgG antibodies (Santa Cruz)

### 2.5 RNA extraction, cDNA synthesis, and real-time PCR

HepG2 cells cultured in DMEM supplemented with 0 mM, 5.6 mM, or 25 mM glucose were treated with BSA or AGEs for 24 hours. Total RNA was extracted using Trizol (Invitrogen) according to manufacturer's instructions. Total RNA was reverse transcribed to cDNA using the PrimeScriptTM RT reagent Kit (Takara Bio Inc., Japan). Real-time PCR analysis was performed using the SYBR green Premix Ex TaqTM kit (Takara Bio Inc., Japan) according to the manufacturer's instructions. The forward and reverse primers set for ChREBP total were 5′-AACTGGAAGTTCTGGGTGTTC-3′ and 5′-AGGGAGTTCAGGACAGTTGG-3′, respectively. The forward and reverse primers for ChREBP-α were 5′-AGTGCTTGAGCCTGGCCTAC-3′ and 5′-TTGTTCAGGCGGATCTTGTC-3′, respectively. The forward and reverse primers for ChREBP-β were 5′- AGCGGATTCCAGGTGAGG-3′ and 5′-TTGTTCAGGCGGATCTTGTC-3′, respectively. The forward and reverse primers for ACC were 5′-GAAAACATCCCGTACCTTCTTC-3′ and 5′-AAGCCTTCACTGTTCCTTCC-3′, respectively. The forward and reverse primers for SCD1 were 5′- GTCCTTATGACAAGAACATTAGCC-3′ and 5′-AATCAATGAAGAATGTGGTGAAG-3′, respectively. The forward and reverse primers for SLC6A9 were 5′-TCTCCCGCCATCATCTTC-3′ and 5′-TTTTCAAACGCTGGAGGAG-3′, respectively. The forward and reverse primers set for GAPDH were 5′-GAGTCAACGGATTTGGTCGT-3′ and 5′-GACAAGCTTCCCGTTCTCAG-3′, respectively. GAPDH was used as the endogenous control.

### 2.6 Analysis of cellular ROS levels

ROS levels were analyzed using the following 2 methods. For fluorescent microscopy, cells were treated with 10 μM dihydroethidium (DHE), incubated for 1 hour at 37°C, and then washed 3 times with PBS. The fluorescence of HepG2 cells was observed using fluorescent microscopy. For flow cytometry, ROS levels were measured using 2,7-dichlorodihydrofluorescein diacetate (DCFH-DA, Sigma-Aldrich). HepG2 cells were washed and incubated with 10 μM DCFH-DA for 40 minutes. HepG2 cells were trypsinized, harvested, washed twice with PBS, and directly collected before the immediate detection of the mean fluorescence intensity (MFI) of DCF to measure cellular ROS levels (excitation 490 nm, emission 520 nm).

### 2.7 Statistical analysis

Each independent experiment was performed at least in triplicate. The data shown were expressed as the means ± standard deviation (SD) and statistically analyzed using the Statistical Package for the Social Sciences, version 16.0 (SPSS Inc.). For the comparison of 2 groups, a student's *t* test was used. A value of *P* < .05 was considered statistically significant.

## 3 Results

### 3.1 AGEs treatment increases S-phase population and inhibits apoptosis in liver cancer cells

**Figure 1:**
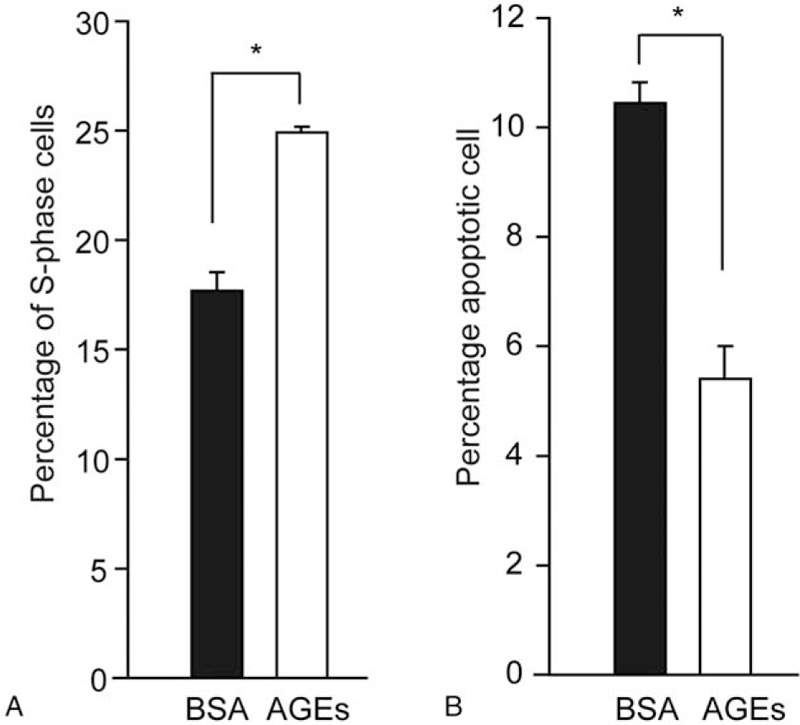
200 mg/L AGEs treatment for 24 hours increased S-phase population (A) and reduced apoptosis (B) in HepG2 cells cultured in the 0 mM glucose medium. BSA served as the negative control for AGEs treatment and ∗ indicated *P* < .05. AGEs = advanced glycation end products, BSA = bovine serum albumin.

We previously reported that AGEs increased human liver cancer HepG2 cell proliferation when compared to the BSA control-treated cells under the 0 mM and 5.6 mM glucose conditions.^[15]^ We chose to study HepG2 cells because ChREBP and RAGE were expressed in this liver cancer cell line.^[29,30]^ To further determine whether AGEs could induce HepG2 cell proliferation, we labeled AGEs-treated HepG2 cells with BrdU and used flow cytometry to observe cell cycle. The percentage of S-phase cells were increased in HepG2 cells cultured in 0 mM glucose medium treated with 200 mg/L AGEs for 24 hours (Fig. 1A). To further assess cell apoptosis effect of AGEs in HepG2 cells, we compared the percentages of apoptotic HepG2 cells which were cultured in 0 mM glucose conditions with either BSA or AGEs. In HepG2 cells which were cultured in 0 mM glucose conditions, compared with the control, AGEs treatment reduced HepG2 cells apoptosis (Fig. 1B). These data showed that AGEs could increase S-phase population and inhibit apoptosis in liver cancer cells.

### 3.2 AGEs increase ChREBP mRNA and protein expression in liver cancer cells

**Figure 2:**
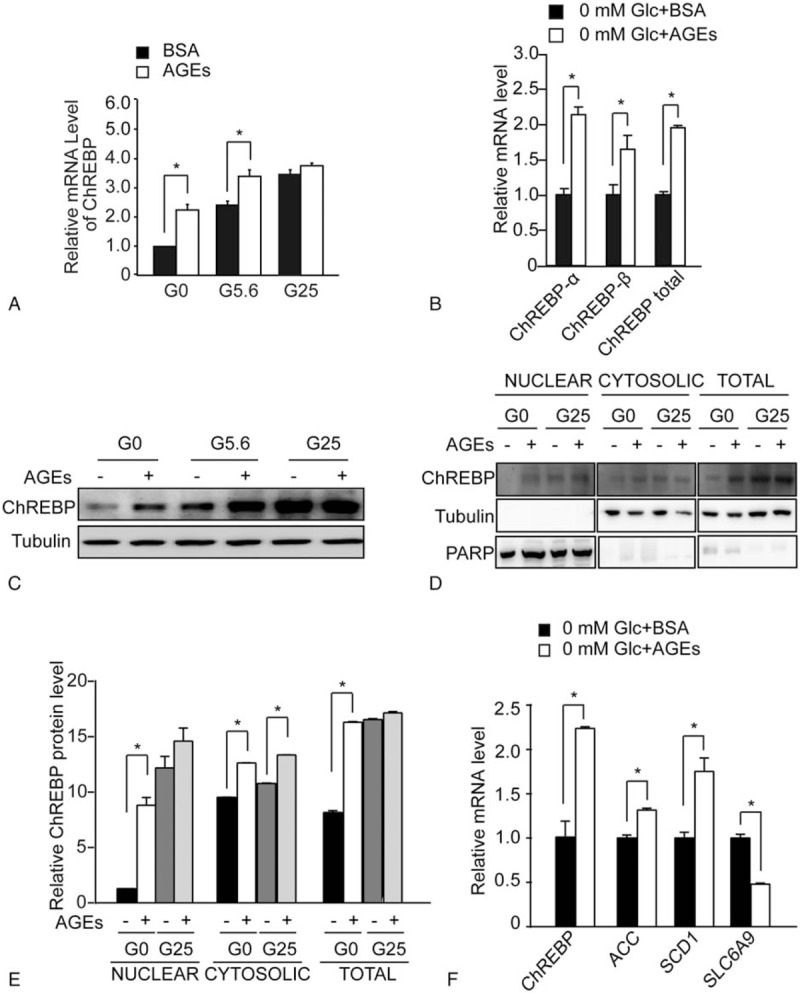
AGEs increased ChREBP expression and promoted ChREBP nuclear translocation in HepG2 cells. (A) Real-time PCR analysis of ChREBP mRNA levels in HepG2 cells treated with either 200 mg/L BSA or 200 mg/L AGEs under 0 mM (G0), 5.6 mM (G5.6), or 25 mM (G25) glucose conditions. Asterisk (∗) indicates *P* < .05 when comparing AGEs- and BSA-treated samples. (B) Real-time PCR analysis of mRNA levels of ChREBP-α, ChREBP-β,and ChREBP total in HepG2 cells treated with either 200 mg/L BSA or 200 mg/L AGEs under 0 mM glucose conditions. Asterisk (∗) indicates *P* < .05 when comparing AGEs- and BSA-treated samples. (C) Western blot analysis of total protein extracts of HepG2 cells treated with BSA (–) or AGEs (+) for 24 hours under 0 mM (G0), 5.6 mM (G5.6), or 25 mM (G25) glucose conditions. The tubulin blot was used as a loading control. (D) Western blot analysis of nuclear, cytosolic, and total protein extracts of HepG2 cells treated with BSA (–) or AGEs (+) for 24 hours under 0 mM or 25 mM glucose conditions. The poly ADP-ribose polymerase blot was used as a loading control for the nuclear fraction. The tubulin blot was used as a loading control for the cytosolic and total protein fraction. (E) Quantitative results of the western blot analysis in B. Asterisk (∗) indicates *P* < .05 compared with BSA-treated samples (–) under the 0 mM glucose condition (G0). (F) Real-time PCR analysis of mRNA levels of ChREBP target genes including ACC, SCD1, and SLC6A9 in HepG2 cells treated with either 200 mg/L BSA or 200 mg/L AGEs under 0 mM glucose conditions. Asterisk (∗) indicates *P* < .05 when comparing AGEs- and BSA-treated samples. AGEs = advanced glycation end products, BSA = bovine serum albumin, ChREBP = carbohydrate responsive element binding protein, PCR = polymerase chain reaction.

We have reported that AGEs promoted ChREBP expression and activity in colorectal cancer cells.^[15]^ Similarly, we investigated whether AGEs changed ChREBP expression in HepG2 cells by treating cells with different concentration of glucose conditions supplemented with either AGEs or BSA for 24 hours. Under 0 mM and 5.6 mM glucose medium, ChREBP mRNA levels were higher after AGEs treatment compared with control cells (Fig. 2A). However, we found that AGEs treatment with 25 mM glucose medium did not increase ChREBP mRNA levels compared with BSA-treated cells (Fig. 2A). Moreover, under 0 mM glucose condition, AGEs treatment increased ChREBP-α, ChREBP-β, and ChREBP total mRNA levels compared with control cells (Fig. 2B). Under 0 and 5.6 mM glucose medium, the protein level of ChREBP increased in AGEs-treated HepG2 cells (Fig. 2C). The ChREBP protein level greatly increased in HepG2 cells which were cultured in 25 mM glucose medium, compared with 0 mM and 5.6 mM glucose conditions (Fig. 2C). Consistent with the real-time PCR results, AGEs treatment did not increase the ChREBP expression under the 25 mM glucose medium in HepG2 cells (Fig. 2C).

It was reported that glucose could promote ChREBP to translocate to the nucleus.^[25]^ We performed nuclear and cytosolic fractionation to investigate the nuclear ChREBP protein level in AGEs-treated HepG2 cells. The study was carried out under 0 mM and 25 mM glucose conditions. Without AGEs treatment, 25 mM glucose increased the total, cytosolic and nuclear ChREBP protein levels compared with the 0 mM glucose condition (Fig. 2D). Under the 0 mM glucose condition, AGEs increased the total, cytosolic, and nuclear ChREBP protein levels compared with BSA treatment (Fig. 2D and E). AGEs greatly enhanced nuclear ChREBP expression under the 0 mM glucose condition (Fig. 2D and E). Under the 25 mM glucose condition, AGEs did not further increase the total and nuclear ChREBP levels while moderately increase the cytosolic ChREBP level (Fig. 2D and E). These findings suggest that AGEs greatly increase nuclear ChREBP protein levels under the glucose-free condition.

We then analyzed the mRNA levels of ChREBP target genes. We found that mRNA expression of ChREBP-activated target genes including ACC and SCD1 increased in AGEs-treated HepG2 cells compared with controls (Fig. 2F). In contrast, AGEs treatment decreased mRNA expression of ChREBP-repressed target gene such as SLC6A9 in HepG2 cells (Fig. 2F).

### 3.3 AGEs stimulate intracellular ROS production in liver cancer cells

**Figure 3:**
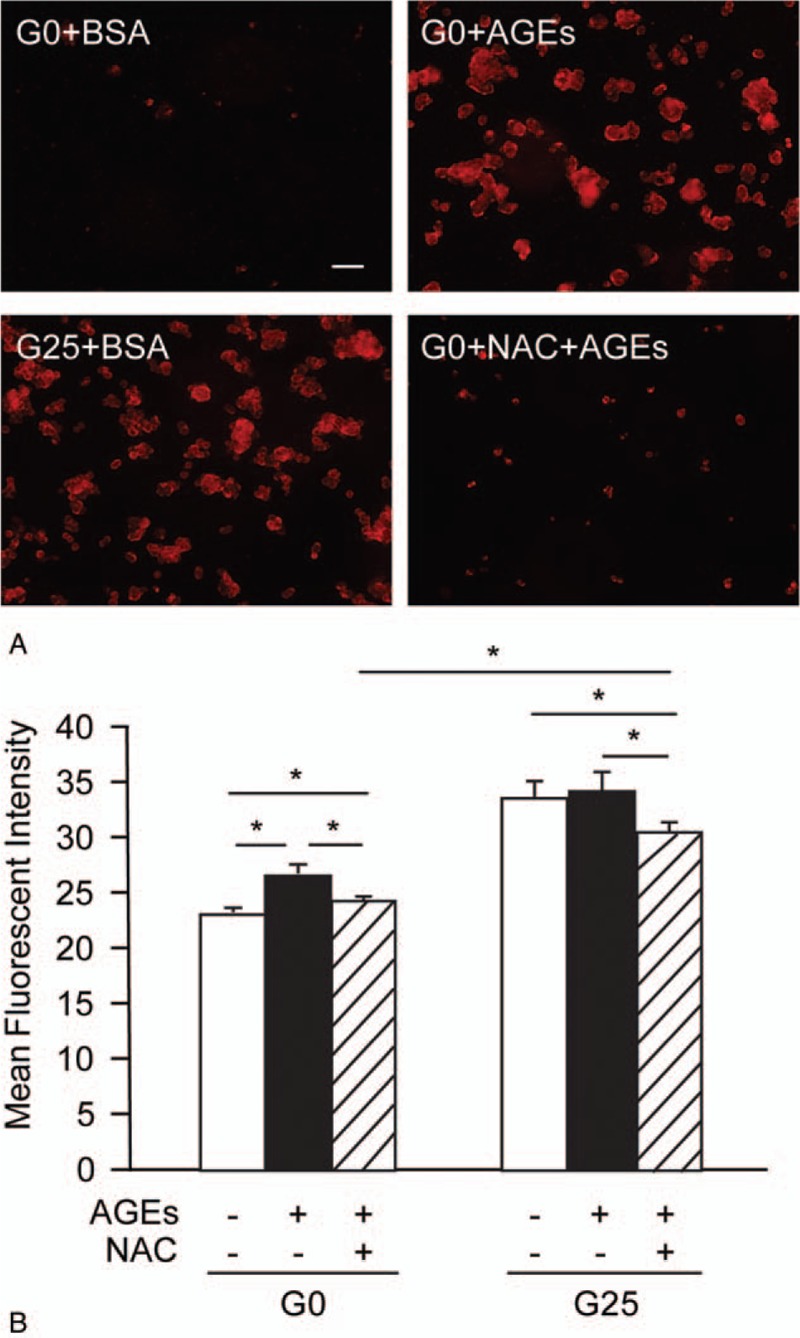
AGEs stimulated ROS generation in HepG2 cells. (A) ROS production detected by a DHE probe using fluorescence microscopy in HepG2 cells treated with either 200 mg/L BSA or 200 mg/L AGEs under 0 mM (G0) or 25 mM (G25) glucose conditions for 24 hours. The scale bar is 20 μm. (B) Intensity of DCFH fluorescence for HepG2 cells with or without 100 μM NAC treatment before being cultured in either the BSA (−) or AGEs (+) under 0 mM (G0) or 25 mM (G25) glucose conditions for 24 hours. Asterisk (∗) indicates *P* < .05. The difference between the 2 shading bars is also statistically significant. AGEs = advanced glycation end products, BSA = bovine serum albumin, DCFH = dichlorodihydrofluorescein diacetate, DHE = dihydroethidium, ROS = reactive oxygen species.

AGEs enhance intracellular ROS generation in diabetic complications such as atherosclerosis and nephropathy.^[3,18,19]^ However, it remains unclear whether AGEs change intracellular ROS levels in cancer cells. Therefore, we examined ROS generation in AGEs-treated HepG2 cells by a DHE probe using fluorescence microscopy. Either 200 mg/L AGEs or 25 mM glucose treatment increased ROS production in HepG2 cells cultured in the glucose-free medium, when compared to control cells (Fig. 3A). Moreover, pretreatment of antioxidant NAC significantly decreased intracellular fluorescence in the AGEs-treated groups, suggesting that antioxidant effectively decreased intracellular ROS levels (Fig. 3A). Similarly, using the DCFH-DA probe to detect ROS production by flow cytometry, we observed that ROS levels increased after AGEs treatment compared with BSA-treated cells under 0 mM glucose conditions (Fig. 3B). In addition, pretreatment of antioxidant NAC significantly decreased the amount of ROS release in the AGEs-treated and 25 mM glucose-treated groups (Fig. 3B). Taken together, our results show that AGEs increase intracellular ROS levels in liver cancer cells.

### 3.4 Antioxidant NAC decreases the induction of ChREBP expression and cell proliferation by AGEs in liver cancer cells

**Figure 4:**
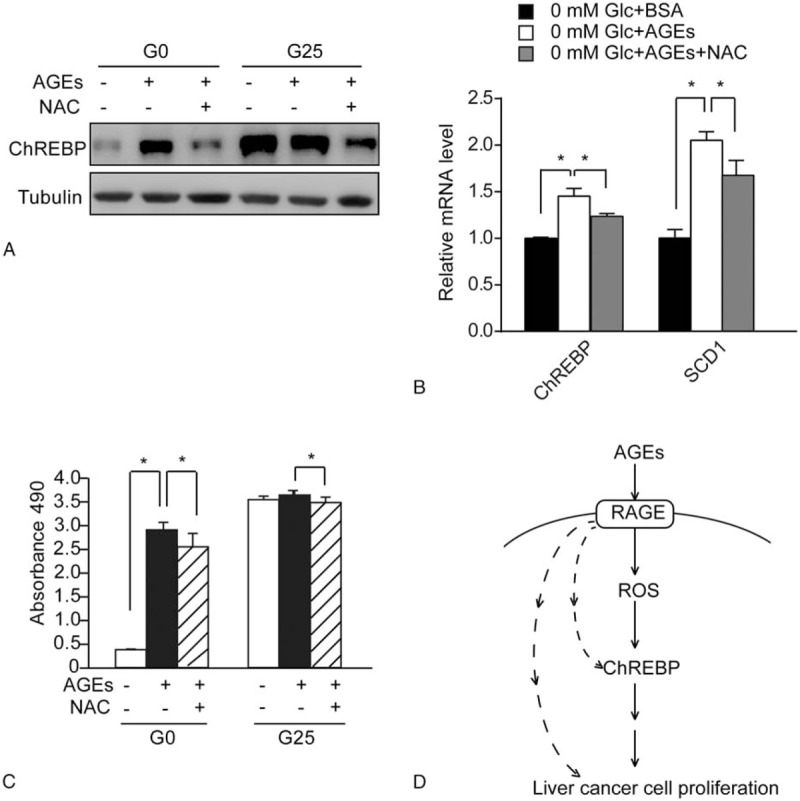
NAC partly reduced the induction of ChREBP expression and cell proliferation in AGEs-treated HepG2 cells. (A) Western blot analysis of total protein extracts of HepG2 cells with or without 100 μM NAC treatment before being cultured in either BSA (−) or AGEs (+) under 0 mM (G0) or 25 mM (G25) glucose conditions for 24 hours. The tubulin blot was used as a loading control. (B) Real-time PCR analysis of mRNA levels of ChREBP and its target gene SCD1 in HepG2 cells treated with either 200 mg/L BSA, 200 mg/L AGEs, or 200 mg/L AGEs and 200 μM NAC under 0 mM glucose conditions. Asterisk (∗) indicates *P* < .05. (C) Analysis of cell proliferation by the MTS reagent in HepG2 cells with or without 100 μM NAC treatment before being cultured in either BSA (−) or AGEs (+) under 0 mM (G0) or 25 mM (G25) glucose conditions for 24 hours. Asterisk (∗) indicates *P* < .05. (D) Schematic model of the AGEs-RAGE-ROS-ChREBP pathway promoting proliferation in human liver cancer cells. The dotted lines indicate other pathways mediating AGEs-RAGE-induced liver cell proliferation. AGEs = advanced glycation end products, BSA = bovine serum albumin, ChREBP = carbohydrate responsive element binding protein, NAC = antioxidant *N*-acetyl cystein, PCR = polymerase chain reaction, RAGE = receptor for advanced glycation end-products, ROS = reactive oxygen species.

Next, we examined whether AGEs acted through ROS generation to increase ChREBP expression and cell proliferation in HepG2 cells. Using Western blot analysis, we found that antioxidant NAC decreased AGEs-induced ChREBP expression (Fig. 4A). This result suggests that AGEs-induced ROS generation is required for increased ChREBP expression. We also noticed that NAC blocked glucose-induced ChREBP expression in HepG2 cells (Fig. 4A). Moreover, NAC treatment reduced AGEs-induced transcriptional activation of ChREBP target gene such as SCD1 (Fig. 4B). These findings suggest that ROS-mediated oxidative stress played an important role in upregulating ChREBP expression and activity in response to AGEs.

We also investigated whether ROS generation played an important role in mediating the increase in cell proliferation by AGEs in HepG2 cells. Pre-treating HepG2 cells with antioxidant NAC decreased AGEs-induced cell proliferation in HepG2 cells (Fig. 4C). Therefore, ROS partly mediates AGEs-induced cell proliferation in HepG2 cells.

## 4 Discussion

In this paper, we have demonstrated that AGEs can act through ROS to induce proliferation in human liver cancer cells. Increased ChREBP expression and activity plays an important role in mediating AGEs/ROS-induced liver cancer cell proliferation (Fig. 4D). Our results not only reveal a novel role of ROS in promoting ChREBP expression, but also suggest that the AGEs-ROS-ChREBP pathway might serve as a new target for the treatment of liver cancer in diabetic patients.

Expression of ChREBP is regulated by various signals including glucose, AGEs, insulin, polyunsaturated fatty acids (PUFA), lipopolysaccharide (LPS), branched-chain amino acids (BCAAs), anoxia, and TGF-β.^[28,31–33]^ Our findings suggest that ROS might regulate ChREBP expression in liver cancer cells. ROS modulate activation of protein kinase C, mitogen-activated protein kinases, and various cytokines and transcription factors such as FOXO, Nrf2, AP-1, and NF-κB.^[3,18,19]^ It will be intriguing to find out the detailed mechanism by which ROS promotes the ChREBP expression. Moreover, suppression of ChREBP activates mitochondrial oxygen consumption and increases ROS production.^[24]^ It is possible ROS and ChREBP regulate each other in a feed-back manner to cooperatively modulate cancer cell metabolism and proliferation.

Our previous findings and this study has both indicated that AGEs and glucose increase ChREBP mRNA and protein levels in cancer cells.^[15]^ One difference is that the effect of AGEs on ChREBP expression depends on the presence of RAGE whereas RAGE seems to play a minor role in glucose-mediated ChREBP induction.^[15]^ We recently reported that HNF-4α played an important role in mediating glucose-induced ChREBP expression.^[34]^ It is worthwhile to find out whether HNF-4α also contributed to AGEs-induced ChREBP expression.

We previously reported that AGEs increased S-phase population and inhibited apoptosis in colorectal cancer cells, leading to enhanced cell proliferation.^[15]^ Here, we observed that liver cancer cells treated with AGEs also showed increased S-phase population and inhibited apoptosis. Suppression of ChREBP not only decreased de novo nucleotide biosynthesis, but also activated p53 signaling in cancer cells.^[24]^ Therefore, AGEs could regulate cell cycle and apoptosis by promoting ChREBP expression, increasing de novo nucleotide biosynthesis and activating p53.

Diabetic patients with some types of cancer such as liver cancer have increased cancer-related mortality, suggesting that the diabetic condition promotes cancer progression.^[35–39]^ Our finding of the AGEs-ROS-ChREBP pathway promoting liver cancer cell proliferation may provide a new explanation for increased liver cancer mortality in diabetic patients.
