# Advanced glycation end products promote ChREBP expression and cell proliferation in liver cancer cells by increasing reactive oxygen species: Erratum

**DOI:** 10.1097/MD.0000000000009318

**Published:** 2017-12-15

In the article, “Advanced glycation end products promote ChREBP expression and cell proliferation in liver cancer cells by increasing reactive oxygen species”^[1]^, which appeared in Volume 96. Issue 33 of *Medicine*, the authors would like to note that Dr. Hanbei Chen, Yakui Li and Yemin Zhu all contributed equally to the article.
